# Interpretability of Paired Pneumococcal Serotype-Specific IgG Testing in Adults With Solid Tumors: A Retrospective Chart-Review Cohort Study

**DOI:** 10.7759/cureus.117519

**Published:** 2026-10-06

**Authors:** Andrew Thake, Jacqueline Squire, Sebastian Fernandez-Bussy, Keith Sacco

**Affiliations:** 1 Internal Medicine, Mater Dei Hospital, Msida, MLT; 2 Allergy/Immunology, Mayo Clinic, Jacksonville, USA; 3 Pulmonary Medicine, Mayo Clinic, Jacksonville, USA

**Keywords:** antibody deficiency, chart review, immunoglobulin replacement, pneumococcal serology, pneumococcal vaccination, secondary hypogammaglobulinemia, solid tumors, specific antibody deficiency, test utilization, vaccine response

## Abstract

Background

Paired pneumococcal serotype-specific immunoglobulin G (IgG) testing may be used when evaluating adults with solid tumors and suspected antibody deficiency, but interpretation depends on vaccine product, vaccination timing, treatment exposures, and clinical context. We assessed how often paired pneumococcal serology records met technical criteria for interpretation and how serologic classification varied according to vaccine product and threshold convention.

Materials and methods

Adults with a solid tumor and at least two pneumococcal serotype-specific IgG panels were identified in the Mayo Clinic Enterprise Data Warehouse. All 108 patient charts were manually reviewed for vaccine product, documented administration between the paired panels, quantitative immunoglobulins, and relevant treatment exposures. A threshold of 1.3 µg/mL was used after the 23-valent pneumococcal polysaccharide vaccine (PPSV23), and after conjugate vaccination, 0.35 µg/mL was used descriptively as a population-level correlate of protection and not as a diagnostic threshold for specific antibody deficiency (SAD). The technical interpretability framework required a known vaccine product, vaccination documented between the paired panels, post-vaccination sampling four to 12 weeks after vaccination, and no major treatment confounder. Technical eligibility for assessment of polysaccharide antibody responsiveness additionally required PPSV23.

Results

Of the 407 patients with eligible solid tumors, 108 had at least two pneumococcal serotype-specific IgG panels and formed the analytic cohort. Vaccine product was identified in 101 patients (93.5%): 60 received PPSV23 and 41 received a conjugate vaccine. Applying a fixed 1.3 µg/mL threshold across vaccine products classified 49 of 108 patients (45.4%) below the specified serologic threshold, whereas descriptive product-specific thresholds classified 31 of 101 patients (30.7%) with a known vaccine product below the corresponding threshold; and 16 conjugate-vaccine recipients were reclassified as meeting the descriptive product-specific threshold. Vaccination was documented between the index panels in 53 patients (49.1%). Across the full cohort, 53 of 108 patients (49.1%) had at least one major treatment confounder, including 22 (20.4%) who received immunoglobulin replacement. Twelve paired-panel records (11.1%) met all four technical criteria; and nine involved PPSV23 and were therefore technically eligible for assessment of polysaccharide antibody responsiveness.

Conclusions

Only nine of 108 paired-panel records (8.3%) met all technical criteria and involved PPSV23. This finding reflects limitations in the interpretability of available testing records and does not estimate the prevalence of SAD. Clinical SAD could not be established because compatible recurrent infection history was not systematically captured and prior pneumococcal vaccination history was incompletely characterized. Clearer documentation of vaccine product and administration date, relevant treatment exposures, and appropriately timed postvaccination testing may improve the interpretability of pneumococcal antibody assessment in clinical practice.

## Introduction

The American Society of Clinical Oncology and the National Comprehensive Cancer Network recommend pneumococcal vaccination for adults with cancer, ideally before systemic therapy begins [1,2]. Vaccination is therefore a routine component of oncology care. When a patient with a solid tumor later presents with recurrent sinopulmonary infections, evaluation may include pneumococcal serotype-specific immunoglobulin G (IgG) testing before and after vaccination. Following an appropriately conducted polysaccharide challenge, paired serotype-specific IgG measurements can contribute to the assessment of polysaccharide antibody responsiveness and, together with the appropriate clinical context, to the evaluation for specific antibody deficiency (SAD).

Interpretation depends on information that is not contained in the serologic result alone. First, protective-threshold conventions differ according to vaccine product and immunologic context. A concentration of 1.3 µg/mL or greater is commonly used after the 23-valent pneumococcal polysaccharide vaccine (PPSV23), whereas 0.35 µg/mL is a population-level correlate of protection used after conjugate vaccination [3,4]. The latter is not a validated diagnostic threshold for SAD in adults, and the two thresholds are therefore not interchangeable. Moreover, conjugate vaccines assess a different immune response from PPSV23 and do not provide an equivalent test of T-independent polysaccharide responsiveness. Second, systemic corticosteroids, cytotoxic chemotherapy, anti-CD20 therapy, and immunoglobulin replacement may confound interpretation of vaccine responses [3,5-8]. In patients receiving immunoglobulin replacement, measured serotype-specific antibodies may partly reflect passively transferred donor antibodies rather than the patient's endogenous response. Third, interpretation depends on the timing of vaccination and serologic testing. Prospective diagnostic-vaccination studies commonly obtain postvaccination samples approximately four to eight weeks after vaccination [9-11].

These clinical and vaccination variables are distinct from the analytical characteristics of the serotype-specific assay itself. Vaccine product, administration timing, relevant treatment exposures, and prior pneumococcal vaccination history may not be represented in the laboratory result and may require review of the clinical record before paired serology can be interpreted in relation to a vaccine challenge. Serotype-specific assays also have important methodological and platform considerations [12-14], further emphasizing that numerical antibody concentrations should be interpreted in their clinical, vaccination, and analytical context. In patients being evaluated for recurrent infection, uncertainty in these elements may limit conclusions about polysaccharide antibody responsiveness and subsequent assessment for SAD.

The primary objective of this study was to determine how often paired pneumococcal serotype-specific IgG records in adults with solid tumors met technical criteria for interpretation after vaccine product, administration timing, and relevant treatment exposures were established by manual chart review. Secondary objectives were to examine how serologic classification varied under commonly used threshold conventions and to determine how many records meeting all technical criteria involved PPSV23 and were therefore technically eligible for assessment of polysaccharide antibody responsiveness. The study evaluated test interpretability rather than the prevalence or clinical diagnosis of SAD.

## Materials and methods

Design and cohort

This retrospective cohort study used the Mayo Clinic Enterprise Data Warehouse, encompassing Mayo Clinic Rochester, Florida, and Arizona, and the Mayo Clinic Health System. Laboratory data spanned January 2012 through July 2026. The Mayo Clinic Institutional Review Board approved the study (IRB application no. 26-003296) with a waiver of informed consent.

A structured laboratory query identified all patients with at least one pneumococcal serotype-specific immunoglobulin G (IgG) panel. Cross-referencing with recorded oncologic diagnoses identified 1,757 patients with cancer and at least one panel. Patients were classified by malignancy type using a keyword algorithm applied to recorded diagnosis text. The retrospective analytic dataset did not retain the complete keyword dictionary or sufficient information to validate the classification algorithm independently. Hematologic malignancies were excluded to reduce biological heterogeneity because these disorders and their treatments are associated with different prevalences and mechanisms of secondary antibody deficiency compared with solid tumors [5-7]. This exclusion was intended to define a more clinically coherent solid-tumor cohort rather than to imply that antibody deficiency in hematologic malignancy arises exclusively from the malignancy or its treatment. Non-melanoma skin cancers and in situ lesions were also excluded. This yielded 407 patients with eligible solid tumors, of whom 108 had at least two pneumococcal serotype-specific IgG panels and formed the analytic cohort (Figure 1A).

**Figure 1:**
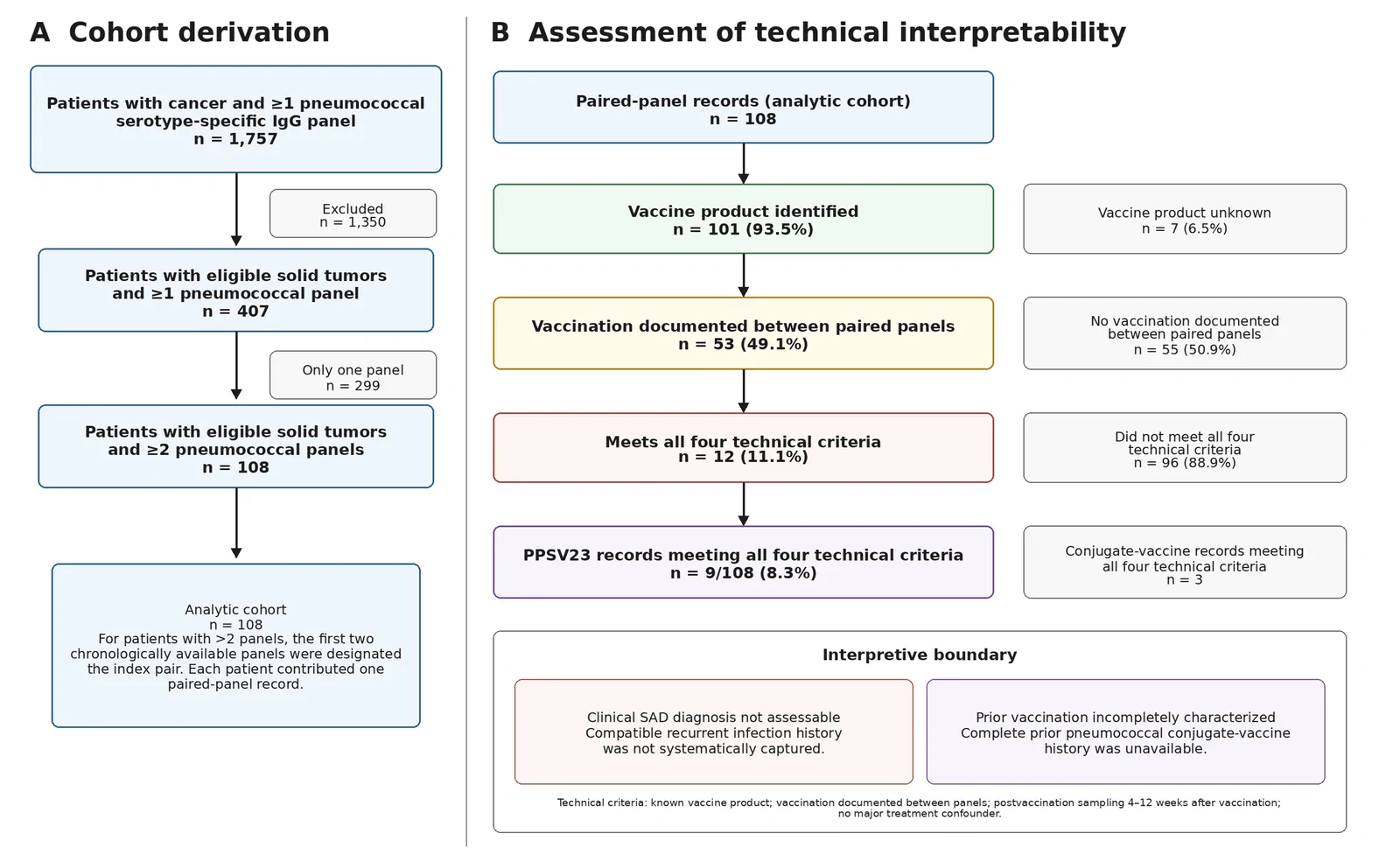
Cohort derivation and assessment of technical interpretability (A) Derivation of the analytic cohort from patients with cancer who had pneumococcal serotype-specific IgG testing. For patients with more than two available panels, the first two chronologically available panels were designated the index pair, and each patient contributed one paired-panel record; (B) Assessment of paired-panel records according to the technical criteria used in this analysis: known vaccine product, vaccination documented between paired measurements, postvaccination sampling four to 12 weeks after vaccination, and absence of a major treatment confounder. Twelve of 108 records met all four technical criteria; nine involved 23-valent pneumococcal polysaccharide vaccine (PPSV23) and were therefore technically eligible for assessment of polysaccharide antibody responsiveness. These records do not establish a clinical diagnosis of specific antibody deficiency (SAD), because compatible recurrent infection history was not systematically captured and prior pneumococcal vaccination history was incompletely characterized.

For patients with more than two available panels, the first two chronologically available panels were designated the index pair. Each patient therefore contributed a single paired-panel record to the primary analysis. Vaccine administration and treatment exposures were subsequently assessed in relation to this index pair by manual chart review. This chronological approach avoided post hoc selection of pairs according to vaccine documentation or observed serologic response and ensured that patients with multiple measurements did not contribute disproportionate weight to the analysis.

Laboratory measurements

Pneumococcal serotype-specific IgG results were obtained from the Enterprise Data Warehouse and were reported in µg/mL. The retrospective analytic dataset did not retain sufficient assay-level metadata to consistently determine the testing platform, lower limit of quantification, panel composition, or platform changes across the 2012-2026 study period. Consequently, no retrospective harmonization across assay platforms was performed.

The study was therefore designed to evaluate the clinical interpretability of paired pneumococcal serology as documented in the available record rather than analytical performance or agreement between laboratory platforms. Potential temporal variation in assay methods over the extended study period remains a source of uncertainty and was considered when interpreting longitudinal comparisons.

Chart abstraction

All 108 charts were manually reviewed by a single trained abstractor using a structured abstraction template. Abstracted variables included pneumococcal vaccine product and administration date; whether vaccination occurred within or outside the Mayo Clinic system; the documentary source supporting the vaccine record; quantitative immunoglobulin values not available in the structured extract; and exposure to systemic corticosteroids, cytotoxic chemotherapy, anti-CD20 therapy, immune checkpoint inhibitors, radiotherapy, and immunoglobulin replacement. Systemic corticosteroid exposure was defined as ≥10 mg/day prednisone equivalent for ≥four weeks.

Corticosteroid, cytotoxic chemotherapy, anti-CD20 therapy, immune checkpoint inhibitor, and radiotherapy exposures were ascertained within 12 months of the first index panel, whereas immunoglobulin replacement was ascertained within six months. These intervals were operational ascertainment windows intended to identify treatment exposures that could complicate interpretation of vaccine-associated serologic measurements. They were not intended to imply that all exposures within a given window exerted equivalent biological effects or that an exposure remained immunologically active throughout the entire interval. More granular timing of individual treatment exposures relative to vaccination and post-vaccination serologic testing was not consistently available in the analytic dataset and therefore could not be incorporated into the technical interpretability framework.

For assessment of technical interpretability, systemic corticosteroids, cytotoxic chemotherapy, anti-CD20 therapy, and immunoglobulin replacement were treated as major treatment confounders. Immune checkpoint inhibitor and radiotherapy exposures were recorded descriptively but were not used to classify a paired-panel record as technically uninterpretable. This framework was intended to identify records with major potential competing influences on measured antibody concentrations rather than to establish a causal effect of any individual treatment on vaccine responsiveness. Uncertain or undocumented fields were retained as missing and were not imputed.

Chart abstraction was performed by a single reviewer without duplicate independent abstraction or formal assessment of inter-rater agreement.

Definitions and study endpoints

The primary endpoint was technical interpretability of the paired pneumococcal serotype-specific IgG record. A paired-panel record was considered technically interpretable when four conditions were met: the vaccine product was known; vaccine administration was documented between the paired measurements; the post-vaccination sample was obtained four to 12 weeks after vaccination; and no major treatment confounder was identified within the ascertainment windows described above. Systemic corticosteroids, cytotoxic chemotherapy, anti-CD20 therapy, and immunoglobulin replacement were considered major treatment confounders for this purpose. Radiotherapy and immune checkpoint inhibitor exposure were recorded descriptively but were not used to determine technical interpretability.

The four-to-12-week interval was the operational post-vaccination window used in this retrospective analysis. This interval is broader than the four-to-eight-week interval commonly used for assessment of pneumococcal vaccine responses [9-11] and should be interpreted as a pragmatic technical criterion rather than as evidence that measurements obtained throughout this interval are diagnostically equivalent.

Serologic classification was a secondary, descriptive endpoint. For records involving PPSV23, a below-threshold profile was defined as fewer than 50% of measured serotypes reaching 1.3 µg/mL. For conjugate-vaccine records, 0.35 µg/mL was used only as a descriptive population-level correlate for comparison [3,4]; it was not considered a validated diagnostic threshold for SAD in adults. A separate analysis applying 1.3 µg/mL across vaccine products was retained to demonstrate the classification generated by a product-agnostic threshold. Accordingly, conjugate-vaccine findings are described as serotypes or profiles below the specified serologic threshold rather than as impaired polysaccharide vaccine responses.

A paired-panel record meeting all four technical criteria and involving PPSV23 was classified as technically eligible for assessment of polysaccharide antibody responsiveness. This designation describes the technical characteristics of the available testing record and does not establish a clinical diagnosis of SAD. Compatible recurrent infection history was not systematically available, and prior pneumococcal vaccination history, including previous conjugate-vaccine exposure, was incompletely characterized. Clinical SAD prevalence therefore could not be determined in this study.

Quantitative immunoglobulins were summarized using the laboratory reference ranges available for the study: IgG 700-1,600 mg/dL, IgA 70-400 mg/dL, and IgM 40-230 mg/dL. IgA and IgM were retained as descriptive measures and to permit comparison with historical definitions that incorporated normal quantitative immunoglobulin concentrations. Missing IgM measurements were not considered sufficient to exclude contemporary assessment of SAD; rather, they limited retrospective classification according to historical definitions requiring additional normal quantitative immunoglobulin parameters. No serologic or quantitative-immunoglobulin category was treated as a clinical diagnosis of SAD.

All paired-panel records were retained in the analytic denominator regardless of documented ordering intent. Ordering intent was not available in a consistently retrievable field, and restricting the analysis to records with explicitly documented diagnostic intent would have selected patients according to documentation completeness, one of the phenomena under investigation. The analysis therefore addressed a pragmatic question: whether paired pneumococcal serology present in the clinical record contained sufficient vaccine, timing, and treatment information to support technical interpretation. Absence of a documented vaccination between paired panels was interpreted only as absence of documentation and not as evidence that vaccination did not occur.

Statistical analysis

Continuous variables were summarized as medians with interquartile ranges and categorical variables as counts and percentages. Independent groups were compared using the Mann-Whitney U test for continuous variables and the chi-square or Fisher exact test for categorical variables, as appropriate. Associations between continuous or ordinal variables were assessed using Spearman rank correlation.

The association between calendar era and classification below the specified serologic threshold was evaluated using logistic regression adjusted for conjugate vaccine receipt. Effect estimates are reported with 95% confidence intervals where available. Inferential analyses were exploratory, and no adjustment was made for multiple comparisons. Accordingly, P values were interpreted alongside effect estimates and confidence intervals and in the context of the exploratory nature of the analyses. All tests were two-sided, with P<0.05 used as the conventional threshold for statistical significance.

Analyses were performed using Python version 3.12 (SciPy version 1.14 and statsmodels version 0.14; Python Software Foundation, Fredericksburg, VA, US) and R version 4.4 (R Foundation for Statistical Computing, Vienna, Austria).

## Results

Cohort characteristics

Among 1,757 patients with a cancer diagnosis and at least one pneumococcal serotype-specific IgG panel, 1,096 (62.4%) had a hematologic malignancy alone and 208 (11.8%) had both hematologic and solid malignancies. An additional 46 patients (2.6%) were excluded because they had only non-melanoma skin cancer or carcinoma in situ, a secondary or unknown primary tumor, or an unclassifiable diagnosis. This yielded 407 patients with eligible solid tumors, of whom 299 had only one pneumococcal panel, and 108 had at least two panels and formed the analytic cohort.

The median age of the analytic cohort was 64 years (interquartile range or IQR, 56-72 years), 73 (67.6%) were female patients, and breast cancer was the most common malignancy (30/108, 27.8%). The median interval between the two index panels was 97 days (IQR, 49-223 days), with 46 patients (42.6%) having an index panel-to-panel interval of four to 12 weeks (Table 1).

**Table 1:** Serologic classification according to the threshold approach The fixed-threshold approach applied a threshold of 1.3 µg/mL across vaccine products. The product-specific analysis used the specified threshold according to vaccine product. The threshold applied to conjugate vaccine recipients was used descriptively and should not be interpreted as a validated diagnostic threshold for specific antibody deficiency in adults. Classification below a serologic threshold does not establish impaired immune competence or a clinical diagnosis of specific antibody deficiency. Seven patients with an unknown vaccine product were not included in the product-specific analysis. NA, not applicable; PPSV23, 23-valent pneumococcal polysaccharide vaccine.

Serologic classification	Fixed 1.3 µg/mL threshold	Product-specific threshold
Patients evaluable, n	108	101
Below specified serologic threshold, n (%)	49 (45.4)	31 (30.7)
Meeting specified serologic threshold, n (%)	59 (54.6)	70 (69.3)
PPSV23 recipients below threshold, n/N (%)	26/60 (43.3)	26/60 (43.3)
Conjugate vaccine recipients below threshold, n/N (%)	21/41 (51.2)	5/41 (12.2)
Conjugate vaccine recipients reclassified as meeting threshold, n	NA	16

This panel-to-panel interval is distinct from the vaccination-to-postvaccination sampling interval used in the technical interpretability criteria. Compared with the 299 patients with only one panel, patients in the analytic cohort had similar age and sex distributions but a lower median serum IgG concentration (690 versus 828 mg/dL; P=0.010).

Treatment exposure

Fifty-three patients (49.1%) had at least one major treatment confounder within the operational ascertainment windows, and 15 (13.9%) had two or more (Table 2).

**Table 2:** Technical interpretability of paired-panel records The four technical criteria were known vaccine product, vaccine administration documented between the index panels, postvaccination sampling four to 12 weeks after vaccination, and absence of a major treatment confounder. Records meeting all four criteria and involving 23-valent pneumococcal polysaccharide vaccine (PPSV23) were considered technically eligible for assessment of polysaccharide antibody responsiveness. Technical eligibility does not establish a clinical diagnosis of specific antibody deficiency. Clinical infection history was not systematically captured, and prior pneumococcal vaccination history was incompletely characterized.

Technical interpretability category	No. (%) (N = 108)
Met all four technical criteria	12 (11.1)
Met three technical criteria	36 (33.3)
Met two technical criteria	42 (38.9)
Met one technical criterion	15 (13.9)
Met no technical criteria	3 (2.8)
Met all four technical criteria and involved PPSV23	9 (8.3)
Met all four technical criteria and involved a conjugate vaccine	3 (2.8)
PPSV23 records meeting all four criteria that were below the 1.3 µg/mL threshold	4/9 (44.4)

Systemic corticosteroid exposure was documented in 26 patients (24.1%), cytotoxic chemotherapy in 18 (16.7%), anti-CD20 therapy in five (4.6%), and immunoglobulin replacement within six months of the first index panel in 22 (20.4%).

Among the 53 patients with vaccine administration documented between the index panels, 25 (47.2%) had at least one major treatment confounder and 28 (52.8%) had none. Radiotherapy was recorded in 27 patients (25.0%) and immune checkpoint inhibitor exposure in six (5.6%). These exposures were recorded descriptively and were not included among the major treatment confounders used in the technical interpretability framework.

Eight of the 22 patients receiving immunoglobulin replacement had serum IgG and IgA concentrations within the laboratory reference ranges. These findings alone would not establish SAD because immunoglobulin replacement may affect measured IgG concentrations and compatible clinical infection history was not systematically available.

Effect of serologic threshold selection

Using a fixed threshold of 1.3 µg/mL across all vaccine products, 49 of 108 patients (45.4%) were classified below the specified serologic threshold. When product-specific serologic thresholds were applied descriptively, 31 of 101 patients with a known vaccine product (30.7%) were classified below the corresponding threshold, including 26 of 60 PPSV23 recipients (43.3%) and five of 41 conjugate vaccine recipients (12.2%) (Table 1).

Sixteen conjugate vaccine recipients who were classified below the fixed 1.3 µg/mL threshold were reclassified as meeting the descriptive product-specific threshold.

Classification below the product-specific serologic threshold was less frequent among conjugate vaccine recipients than among PPSV23 recipients (5/41 (12.2%) versus 26/60 (43.3%); P<0.001). This comparison reflects differences in the threshold conventions applied to the two vaccine types and should not be interpreted as evidence of differences in immune competence or as establishing a clinical diagnosis of SAD.

Technical interpretability of paired-panel records

Twelve of 108 paired-panel records (11.1%) met all four technical criteria; 36 (33.3%) met three criteria, 42 (38.9%) met two, 15 (13.9%) met one, and three (2.8%) met none (Table 2 and Figure 1B). Of the 12 records meeting all four criteria, nine involved PPSV23 and three involved a conjugate vaccine. Thus, only nine of 108 paired-panel records (8.3%) met all technical criteria and involved PPSV23, making them technically eligible for assessment of polysaccharide antibody responsiveness.

Among these nine PPSV23 records, four (44.4%) were classified below the 1.3 µg/mL serologic threshold. Two of the nine had documented IgG and IgA concentrations within the laboratory reference ranges, but neither had an available IgM measurement. These findings describe serologic and quantitative immunoglobulin patterns only. Clinical SAD could not be established because compatible recurrent infection history was not systematically captured and prior pneumococcal vaccination history was incompletely characterized.

Subgroup analyses

Classification below the product-specific serologic threshold occurred at similar frequencies among patients with IgG concentrations below 700 mg/dL (15/50, 30.0%) and those with concentrations of 700-1,600 mg/dL (14/44, 31.8%; P=1.00). This comparison was restricted to the 94 patients with both a known vaccine product and an IgG concentration at or below the upper limit of the laboratory reference range. Serum IgG concentration showed a weak positive correlation with the proportion of serotypes meeting the specified serologic threshold (Spearman ρ=0.19; P=0.065).

Among patients with a known vaccine product, classification below the product-specific serologic threshold occurred in 10 of 21 patients receiving immunoglobulin replacement (47.6%) and 21 of 80 not receiving replacement (26.2%; OR, 2.55; P=0.068). One of the 22 patients receiving immunoglobulin replacement had no identified vaccine product and was therefore excluded from this comparison.

In exploratory calendar-era analyses, classification below the fixed 1.3 µg/mL threshold was more frequent when the first index panel occurred in 2022 or later than before 2022 (55.6% versus 31.1%; P=0.018). In an analysis using product-specific thresholds and adjusting for conjugate vaccine receipt, the association with calendar era remained (adjusted OR, 2.96; 95% CI, 1.09-7.99; P=0.033).

## Discussion

The principal finding of this study is the limited technical interpretability of paired pneumococcal serotype-specific IgG testing in this retrospective solid-tumor cohort. Although 108 patients had at least two serotype panels, only 12 paired-panel records met all four technical criteria used in this analysis, and only nine (8.3%) involved PPSV23 and were therefore technically eligible for assessment of polysaccharide antibody responsiveness. This finding quantifies limitations in the interpretability of available testing records; it does not estimate the prevalence of SAD or imply that the remaining patients underwent failed diagnostic challenges. Clinical SAD could not be established because compatible recurrent infection history was not systematically captured and prior pneumococcal vaccination history was incompletely characterized. Earlier work has also evaluated pneumococcal vaccine antibody responses in patients with solid tumors and lymphomas [15].

Threshold selection substantially affected serologic classification. Applying a fixed threshold of 1.3 µg/mL across vaccine products classified 49 of 108 patients (45.4%) below the specified threshold, whereas descriptive product-specific thresholds classified 31 of 101 patients with a known vaccine product (30.7%) below the corresponding threshold. Sixteen conjugate vaccine recipients were reclassified as meeting the descriptive product-specific threshold. This change should not be interpreted as correction of a SAD diagnosis. The 0.35 µg/mL threshold used for conjugate vaccine recipients represents a population-level correlate associated with protection and is not a validated diagnostic threshold for SAD in adults. Moreover, conjugate vaccines do not provide the same assessment of T-independent polysaccharide responsiveness as PPSV23. The observed reclassification instead demonstrates how interpretation of pneumococcal serology depends on vaccine product and threshold convention. Previous studies have similarly shown that serotype-specific classification varies according to age, serotype, assay approach, and response definition [9-14].

Treatment exposure represented an additional source of uncertainty. Major treatment confounders were identified in 53 patients (49.1%), including immunoglobulin replacement in 22 (20.4%). Eight patients receiving immunoglobulin replacement had serum IgG and IgA concentrations within the laboratory reference ranges. These measurements alone cannot establish SAD because immunoglobulin replacement may influence measured IgG concentrations and pneumococcal serotype-specific antibodies may include passively transferred donor antibodies. The higher frequency of classification below the product-specific serologic threshold among patients receiving replacement (47.6% versus 26.2%; OR, 2.55; P=0.068) should therefore be interpreted cautiously. The retrospective design, small subgroup, absence of detailed treatment timing, and potential confounding by the underlying indication for replacement preclude attribution of this association to immunoglobulin replacement itself.

Documentation of vaccination was another important limitation. Vaccine administration between the index panels was documented in 53 patients (49.1%); in the remaining 55, no administration was documented between the paired measurements. Absence of documentation does not establish that vaccination did not occur. Administration may have occurred outside the available record, before the index interval, or may not have occurred at all. Similarly, the median panel-to-panel interval of 97 days and the proportion of index panel pairs separated by four to 12 weeks should not be interpreted as vaccination-to-post-vaccination timing. Assessment of vaccine-associated antibody responsiveness requires a documented vaccine exposure and appropriately timed post-vaccination sampling [10,11,16]. These findings therefore illustrate why the presence of two serotype panels alone is insufficient to establish that they constitute a diagnostic vaccine challenge.

Not every paired panel in this cohort was necessarily ordered as part of a deliberate diagnostic challenge. Ordering intent was not available in a consistently retrievable field, and restricting the analysis to records with explicitly documented diagnostic intent would have selected patients partly according to documentation completeness. We therefore retained all paired-panel records and assessed their technical interpretability using the same framework. Accordingly, the 8.3% estimate should be understood as the proportion of available paired-panel records that met all technical criteria and involved PPSV23, rather than as the success rate of intentionally ordered diagnostic challenges.

Quantitative immunoglobulin abnormalities were also common in this selected population. Among the 106 patients with an available IgG measurement, 53 (50.0%) had IgG below 700 mg/dL, while 38 of 108 patients (35.2%) had documented IgG and IgA concentrations within the laboratory reference ranges. These findings indicate that abnormal quantitative immunoglobulin concentrations frequently coexisted with pneumococcal serotype testing. They do not establish the prevalence or cause of secondary antibody deficiency among patients with solid tumors because the cohort was selected for pneumococcal serologic testing and the study could not reliably distinguish pre-existing, malignancy-associated, and treatment-associated abnormalities. The high prevalence of antibody deficiency described in hematologic malignancies provides relevant clinical context [5-7], but those populations differ biologically and therapeutically from the solid-tumor cohort studied here.

Calendar era was associated with serologic classification in exploratory analyses. Classification below the fixed 1.3 µg/mL threshold was more frequent when the first index panel occurred in 2022 or later, and the association remained when product-specific thresholds were used with adjustment for conjugate vaccine receipt (adjusted OR, 2.96; 95% CI, 1.09-7.99; P=0.033). Several factors could potentially contribute to temporal differences, including changing pneumococcal vaccination practices, prior conjugate vaccine exposure, patient selection, and assay-related changes over the 2012-2026 study period. Complete prior pneumococcal vaccination histories and sufficient assay-level metadata were unavailable, preventing these possibilities from being distinguished. The calendar-era association should therefore be considered exploratory rather than evidence of a specific biological or vaccine-sequence effect.

Implications for practice

The findings identify several documentation and testing processes that may improve the interpretability of pneumococcal serotype-specific IgG testing. First, vaccine product and administration date should be documented clearly when serologic assessment of vaccine responsiveness is intended. In this cohort, the vaccine product could not be determined in seven patients, the administration site was undocumented in 22, and vaccination between the index panels was documented in only 53 of 108 patients.

Second, potentially confounding treatment exposures should be considered when interpreting serotype-specific antibody measurements. Systemic corticosteroids, cytotoxic chemotherapy, anti-CD20 therapy, and immunoglobulin replacement were treated as major treatment confounders in this analysis. Immunoglobulin replacement deserves particular consideration because measured total and serotype-specific IgG may be influenced by replacement therapy.

Third, when a diagnostic vaccine challenge is intended, the relationship between vaccination and postvaccination sampling should be documented explicitly. The four-to-12-week interval used in this retrospective analysis was an operational technical criterion and is broader than the four-to-eight-week interval commonly used in diagnostic and vaccine-immunogenicity studies [10,11,16]. Panel-to-panel spacing should not be used as a substitute for documented vaccination-to-postvaccination timing.

Finally, quantitative immunoglobulin measurements provide complementary information when evaluating suspected antibody deficiency. IgG and IgA were available for most patients in this cohort, whereas IgM was measured in only 13 of 108 patients. Missing IgM limits retrospective application of historical definitions incorporating normal IgG, IgA, and IgM, but does not by itself preclude contemporary assessment of SAD [17]. Quantitative immunoglobulin concentrations and functional antibody assessment should therefore be interpreted alongside clinical infection history, vaccination history, and relevant treatment exposures rather than as interchangeable measures.

Strengths and limitations

A principal strength of this study was manual review of all 108 charts, which allowed vaccine product, administration documentation, treatment exposures, and additional quantitative immunoglobulin measurements to be examined beyond the structured laboratory extract. The use of an explicit technical interpretability framework also separated the availability of paired serologic measurements from their suitability for assessment of polysaccharide antibody responsiveness.

Several limitations are important. This was a retrospective study of a selected population of patients with solid tumors who underwent pneumococcal serotype-specific IgG testing; consequently, the observed proportions should not be interpreted as estimates of the prevalence of abnormal serologic responses or SAD among patients with solid tumors generally. Ordering intent was not consistently retrievable, so the cohort included paired panels that may have been obtained for diagnostic assessment, monitoring, or other purposes. Absence of documented vaccination between panels likewise does not establish that vaccination did not occur.

Clinical infection history was not systematically captured, and complete prior pneumococcal vaccination history, including prior conjugate vaccine exposure, was unavailable. Clinical SAD therefore could not be established. IgM was measured in only 13 of 108 patients, limiting retrospective application of historical definitions that incorporated normal concentrations of all major quantitative immunoglobulin classes; however, missing IgM should not itself be interpreted as excluding contemporary SAD assessment.

Treatment exposures were identified using operational ascertainment windows. More granular timing relative to vaccination and post-vaccination serologic testing was not consistently available, and exposures occurring within these windows should not be assumed to have equivalent biological effects. Chart abstraction was performed by a single reviewer without duplicate independent abstraction or formal assessment of inter-rater agreement.

The retrospective analytic dataset also lacked sufficient assay-level metadata to consistently determine testing platform, lower limit of quantification, panel composition, or platform changes across the 2012-2026 study period, and retrospective assay harmonization was therefore not possible. This is particularly relevant to longitudinal and calendar-era comparisons. The nine PPSV23 paired-panel records meeting all technical criteria constitute a small subgroup and do not support precise estimates of the frequency of abnormal polysaccharide antibody responsiveness.

Finally, the cohort was predominantly White (95.4%) and was derived from Mayo Clinic tertiary referral practices and the Mayo Clinic Health System. Differences in patient populations, vaccination practices, laboratory methods, treatment patterns, and documentation workflows may limit the generalizability of the absolute proportions observed here.

## Conclusions

Paired pneumococcal serology in this cohort was frequently limited by incomplete vaccine documentation, sampling timing, and relevant treatment exposures. Only a small subset of paired-panel records met all technical criteria and involved PPSV23. These findings reflect limitations in test interpretability rather than the prevalence of specific antibody deficiency. Serologic results should therefore be interpreted alongside the vaccine product and circumstances of testing. Clearer documentation of vaccination and treatment exposures may improve interpretation in clinical practice. Prospective studies using standardized testing pathways may further clarify the role of pneumococcal serology in this population.
